# How to reprogram microglia toward beneficial functions

**DOI:** 10.1002/glia.23484

**Published:** 2018-09-08

**Authors:** Marta Fumagalli, Marta Lombardi, Pierre Gressens, Claudia Verderio

**Affiliations:** ^1^ Department of Pharmacological and Biomolecular Sciences Università degli Studi di Milano via Balzaretti, 9 ‐20133, Milan Italy; ^2^ IRCCS Humanitas via Manzoni 56, 20089, Rozzano Italy; ^3^ PROTECT, INSERM Université Paris Diderot Sorbonne Paris Cité, 1141 Paris France; ^4^ Centre for the Developing Brain, Department of Perinatal Health and Imaging, Division of Imaging Sciences and Biomedical Engineering King's College London, King's Health Partners, St. Thomas' Hospital London, SE1 7EH United Kingdom; ^5^ CNR Institute of Neuroscience via Vanvitelli 32, 20129 Milan Italy

**Keywords:** beneficial phenotype, metabolism, microglia, miRNA, re‐program

## Abstract

Microglia, brain cells of nonneural origin, orchestrate the inflammatory response to diverse insults, including hypoxia/ischemia or maternal/fetal infection in the perinatal brain. Experimental studies have demonstrated the capacity of microglia to recognize pathogens or damaged cells activating a cytotoxic response that can exacerbate brain damage. However, microglia display an enormous plasticity in their responses to injury and may also promote resolution stages of inflammation and tissue regeneration. Despite the critical role of microglia in brain pathologies, the cellular mechanisms that govern the diverse phenotypes of microglia are just beginning to be defined. Here we review emerging strategies to drive microglia toward beneficial functions, selectively reporting the studies which provide insights into molecular mechanisms underlying the phenotypic switch. A variety of approaches have been proposed which rely on microglia treatment with pharmacological agents, cytokines, lipid messengers, or microRNAs, as well on nutritional approaches or therapies with immunomodulatory cells. Analysis of the molecular mechanisms relevant for microglia reprogramming toward pro‐regenerative functions points to a central role of energy metabolism in shaping microglial functions. Manipulation of metabolic pathways may thus provide new therapeutic opportunities to prevent the deleterious effects of inflammatory microglia and to control excessive inflammation in brain disorders.

## 1 INTRODUCTION

Microglia are self‐renewing, long‐lived cells (Bruttger et al., 2015), which stem from a unique nonhaematopoietic yolk‐sac‐derived cell lineage, as indicated by elegant fate‐mapping studies (Ginhoux et al., 2010; Kierdorf et al., 2013; Schulz et al., 2012). They are critical both for developmental processes such as synaptogenesis and for the maintenance of neural homeostasis (Ekdahl, Kokaia, & Lindvall, 2009; Nikolakopoulou, Dutta, Chen, Miller, & Trapp, 2013; Ribeiro Xavier, Kress, Goldman, Lacerda de Menezes, & Nedergaard, 2015; Sellner et al., 2016; Sierra et al., 2010; Tremblay et al., 2011).

Contributions of microglia to physiological brain function are underlined by functional deficits in neuronal connectivity and neurologic disorders in mice harboring dysfunctional microglia (Prinz & Priller, 2014), mutant for complement or CX_3_CR1 chemokine receptors (Paolicelli et al., 2011; Schafer et al., 2012).

In response to insult or injury, microglia are capable of acquiring diverse and complex phenotypes, allowing them to participate in cytotoxic response as well as in immune regulation or injury resolution (extensively reviewed in (Q. Li & Barres, 2018; Wolf, Boddeke, & Kettenmann, 2017; Colonna & Butovsky, 2017). The deleterious effects of cytotoxic microglia include white and gray matter lesions in brain tissue of both patients with chronic neuroinflammatory diseases such as multiple sclerosis (MS) (Prins et al., 2015) and infants with perinatal inflammation (Dean et al., 2010; Favrais et al., 2011). Conversely, blockage of neuron damage after acute brain injury (Faustino et al., 2011; Imai et al., 2007; Szalay et al., 2016) and promotion of remyelination in mice with myelin lesions (Butovsky et al., 2006; Miron et al., 2013; Olah et al., 2012), reviewed in (Cherry, Olschowka, & O'Banion, 2014), are among supportive functions of pro‐regenerative microglia.

Consolidated evidence indicates that the pro‐inflammatory cytokine interleukin (IL)‐1β, the toll‐like‐receptor (TLR)‐4 agonist lipopolysaccharide (LPS), the TLR‐3 agonist Poly I : C, and tumor necrosis factor‐α (TNFα) are potent inducers of detrimental microglial phenotype in vitro. Functionally, pro‐inflammatory microglia are characterized by microbicidal, antigen‐presenting and immune‐potentiating abilities, through synthesis of nitric oxide (NO), chemokines, and inflammatory cytokines (Amici, Dong, & Guerau‐de‐Arellano, 2017). Conversely, the anti‐inflammatory cytokines IL‐4 and IL‐13, immune complexes, IL‐ 1R ligands, IL‐10, TGF‐β, and glucocorticoids are typical agents which drive microglia toward pro‐regenerative, immunomodulatory or deactivated phenotypes. Pro‐regenerative microglia secrete anti‐inflammatory mediators and express Arg1 instead of inducible NO synthase (iNOS), switching arginine metabolism from production of NO to ornithine and polyamines for collagen and extracellular matrix synthesis. However, detrimental and pro‐regenerative microglia polarization in vitro is highly artificial and does not reflect the real in vivo situation where microglia activation follows a wide spectrum of phenotypes, which may be driven by diverse stimuli, as described for macrophages (Xue et al., 2014).

Using transcriptomic and proteomic approaches (Butovsky et al., 2014; Gautier et al., 2012; Hickman et al., 2013; Krasemann et al., 2017) the molecular signature of microglia in vivo has begun to be defined under physiological and pathological conditions, including amyotrophic lateral sclerosis (ALS) (Chiu et al., 2013), multiple sclerosis (MS), Alzheimer's disease (AD) (Krasemann et al., 2017) or perinatal brain injury (Krishnan et al., 2017). However, our understanding of the molecular mechanisms that govern transition of microglia from detrimental to pro‐regenerative phenotypes both in vitro and in vivo remains limited. Much more is known about cellular pathways controlling polarization in macrophages, the peripheral microglia counterparts, where it is increasingly clear that metabolic activities co‐define their effector state. Specifically, in peripheral macrophages, mitochondrial oxidation is a determinant of pro‐regenerative polarization. Blocking oxidative metabolism abrogates anti‐inflammatory macrophage function without affecting the pro‐inflammatory state (Ghesquiere, Wong, Kuchnio, & Carmeliet, 2014). For a detailed description of both classical and emerging pathways, including metabolic pathways, relevant for macrophage phenotypes we refer the readers to recent excellent reviews (Amici et al., 2017; Diskin & Palsson‐McDermott, 2018).

Despite the genetic profile of microglia differs profoundly from that of macrophages (Yamasaki et al., 2014), recent evidence indicates that energy production in mitochondria may be a determinant of pro‐regenerative/resolving differentiation also in microglia. In fact, reduced mitochondrial oxygen consumption, reduced electron transport, and mitochondrial ATP synthesis caused by deficiency in Clock (Clk)1, a mitochondrial hydroxylase that is necessary for the biosynthesis of ubiquinone (Hekimi, 2013; Lapointe & Hekimi, 2008), has been linked to the acquisition of pro‐inflammatory phenotype by microglia. Importantly, the inflammatory state of Clk1 deficient microglia relies on aerobic glycolysis, as it is abolished when glycolytic metabolism is inhibited by knocking down hexokinase 2 (HK2), a key rate‐limiting enzyme for phosphorylating the glucose (J. J. Gu et al., 2018). However, whether metabolic pathways may take central stage in inducing a repair‐supportive phenotype is not yet established.

In this review, we summarize emerging strategies able to redirect microglia from detrimental to beneficial functions. As we learn more about molecular mechanisms controlling microglia effector functions, a central role of cell metabolism emerges, opening novel approaches to target microglia therapeutically.

## 2 RECEPTOR AND CHANNELS PROMOTING ANTI‐INFLAMMATORY MICROGLIA FUNCTION

Multiple brain's signaling substances converge on microglia to actively maintain or alter their functional state through a variety of neurotransmitter receptors and channels (Domercq, Vazquez‐Villoldo, & Matute, 2013). Here we focus on receptors and channels which are involved in microglia reprogramming toward anti‐inflammatory phenotype with a known mechanism of action.

### 2.1 Histamine and other neurotransmitter receptors

**Figure 1:**
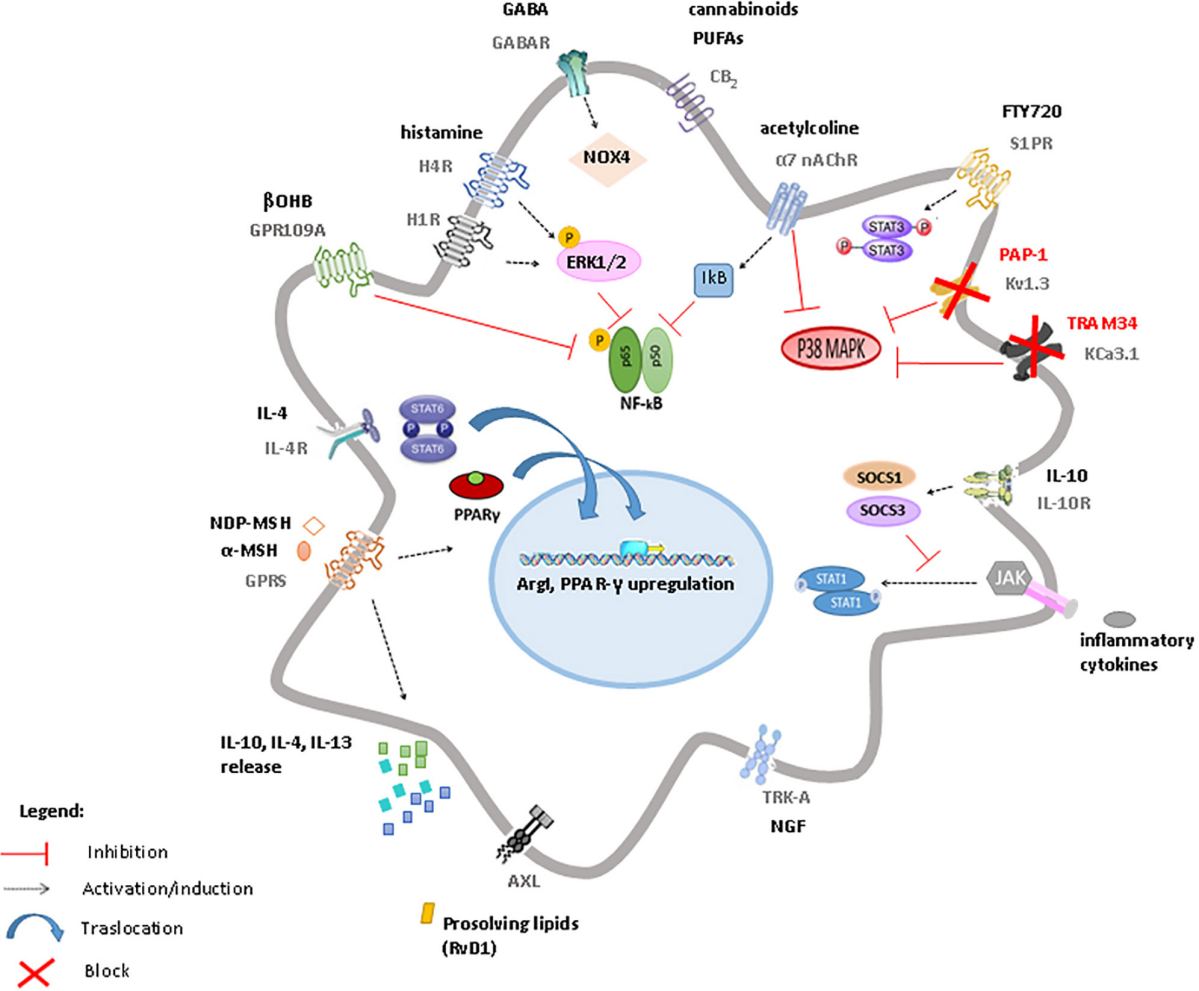
Schematic representation of receptors and signaling pathways mainly contributing to anti‐inflammatory polarization of microglia. Stimulation of histamine receptors H1R and H4R induces persistent ERK1/2 phosphorylation, that leads to pNF‐κB downregulation and reduces the expression of NADPH oxidase 2 (NOX2), dampening pro‐inflammatory responses. Similarly, the endocannabinoid type 2 receptor (CB2) induces sustained ERK1/2 phosphorylation causing pNF‐κB downregulation. GABA receptors are also involved in reducing microglia reactivity through the activation of NOX4. The nicotinic α7 receptor decreases pNF‐κB and its p65 subunit and inhibits p38‐MAPK. Blocking the voltage independent KCa3.1 channels and the voltage‐gated Kv1.3 also results in inhibition of p38‐MAPK pathway and of iNOS and COX expression. IL‐10, upon binding of its receptor, suppresses pro‐inflammatory cytokine production through induction of suppressor of cytokine signalling (SOCS)1 and (SOCS)3 proteins. These proteins, in turn, inhibit the cytokine‐activated Janus kinase (JAK)/ STAT‐1 signaling pro‐inflammatory pathway. The transcription factor signal transducer and activator of transcription (STAT) 3 is instead induced by the activation of sphingosine‐1‐phosphate (S1P) receptors through its ligand FTY720, resulting in attenuation of microglia‐mediated neuroinflammation. NGF, through activation of Trk‐A receptor, reduces cytokine/chemokine secretion, microglia motility, phagocytosis, and degradative pathways. The activation of the tyrosine kinase AXL receptor by pro‐resolving lipid mediators suppresses the pro‐inflammatory microglial phenotype by dampening type I interferon (IFN) signaling. The melanocortins α‐MSH and NDP‐MSH, through G protein‐coupled receptors, enhance PPAR‐γ, and Arg1 expression and promote IL‐10 release. Upon stimulation with IL‐4, IL‐4 receptor (IL‐4R) induces activation of STAT‐6, with consequent increase in Arg1, mannose receptor CD206 and PPAR‐γ expression and with induction (and release) of anti‐inflammatory cytokines like IL‐4, IL‐10, IL‐13. For specific references see text

Histamine has a well‐established role as neuron‐to‐glia alarm signal in the brain and microglia constitutively express all four histamine receptors (H1R, H2R, H3R, and H4R) (W. Hu & Chen, 2017). Apolloni et al. recently demonstrated that histamine counteracts pro‐inflammatory microglia phenotype in the SOD1‐G93A mouse model of ALS. They showed that histamine not only reduces expression of both NADPH oxidase 2 (NOX2), a super‐oxide generating enzyme which forms reactive oxygen species (ROS), and of NF‐κB, the classical transcription factor activated in pro‐inflammatory microglia, but also increases the anti‐inflammatory genes (IL‐10, ARG1, P2Y12, CD163, and CD206) acting through H1R and H4R. Importantly, histamine exerts beneficial action only in inflammatory SOD1‐G93A microglia, which are characterized by decreased H1R levels, while elicits a pro‐inflammatory effect in nontransgenic cells. In support to these findings a recent work showed that histamine deficiency reduces the ramified/surveilling morphology of microglia and the production of anti‐inflammatory factors in the striatum of knockout mice (Frick, Rapanelli, Abbasi, Ohtsu, & Pittenger, 2016). With respect to the signal transduction mechanisms involved downstream of H1R and H4R activation in ALS microglia, Apolloni, and colleagues demonstrated a persistent ERK1/2 phosphorylation, which accounts for downregulation of p‐NF‐κB (Figure 1).

Among other neurotransmitter receptors controlling microglia polarization, recently reviewed by Liu et al. (Liu, Leak, & Hu, 2016), GABA receptors are upregulated in response to brain injuries and act via NOX4 activation (Mead et al., 2012). The endocannabinoid type 2 receptor (CB2) inhibits IL‐6 and TNFα release from microglia (Bisogno & Di Marzo, 2010; Mecha et al., 2018) and increases the suppressive potency of myeloid‐derived suppressor cells. Nicotinic receptors, in particular α7 receptor (Y. Sun et al., 2013; King, Gillevet, & Kabbani, 2017), decreases p‐NF‐κB and its p65 subunit and inhibits p38 mitogen‐activated protein kinase (p38‐MAPK), a key player of inflammatory microglia response (Lawson, Dobrikova, Shveygert, & Gromeier, 2013) (Figure 1), while upregulates antioxidant genes, promoting a pro‐regenerative microglial state (Z. Han et al., 2014). Sphingosine‐1‐phosphate (S1P) receptors downregulate pro‐inflammatory cytokines and enhance pro‐regenerative responses after intracerebral hemorrhage (Marfia et al., 2016; Noda, Takeuchi, Mizuno, & Suzumura, 2013). The S1P receptor ligand FTY720 has been recently shown to attenuate microglia‐mediated neuroinflammation after white matter ischemic injury and to promote oligodendrogenesis via the transcription factor signal transducer activator of transcription (STAT)3 (Qin et al., 2017) (Figure 1), one of the STAT transcription factors that regulate cytokine (El Kasmi et al., 2006) and chemokine (Kwon et al., 2017) production. Of note, S1P is a known activator of mitochondrial function (Nema & Kumar, 2015), suggesting a possible contribution of enhanced respiratory function to microglia phenotypic switch.

### 2.2 Neuropeptide/growth factor receptors

Neuropeptides and neurotrophins acting through specific receptors are able to modulate microglial response by inhibiting the release of inflammatory mediators while favoring development of an alternative activation program (Carniglia et al., 2017; Rizzi et al., 2018). Specifically, the melanocortins α‐MSH and NDP‐MSH, both exerting their actions through G protein‐coupled receptors (GPCRs), were found to induce expression of the metabolic enzyme Arg1 in microglia in the retina (Kawanaka & Taylor, 2011) and in rat primary culture (Carniglia, Durand, Caruso, & Lasaga, 2013). Arg1 induction was accompanied by enhanced expression of the nuclear receptor peroxisome proliferator‐activated receptor gamma (PPAR‐γ) (Szanto et al., 2010), and by enhanced IL‐10 release (Figure 1), known markers of pro‐regenerative microglia (see below).

The classical neurotrophin NGF, through activation of Trk‐A receptors was recently shown to steer microglia toward a neuroprotective and anti‐inflammatory phenotype (Rizzi et al., 2018) (Figure 1), being particularly effective in reverting the pro‐inflammatory state of microglia induced by β‐amyloid. Importantly, the receptor signaling activated by NGF in primary culture and ex vivo not only regulates a number of microglia activities, such as cytokine/chemokine secretion, motility, phagocytosis, and degradative pathways but also controls microglia–neuron interaction, protecting neurons against Aβ‐induced spine alterations and synaptic dysfunction.

### 2.3 Receptors of endogenous immune signals

**Figure 2:**
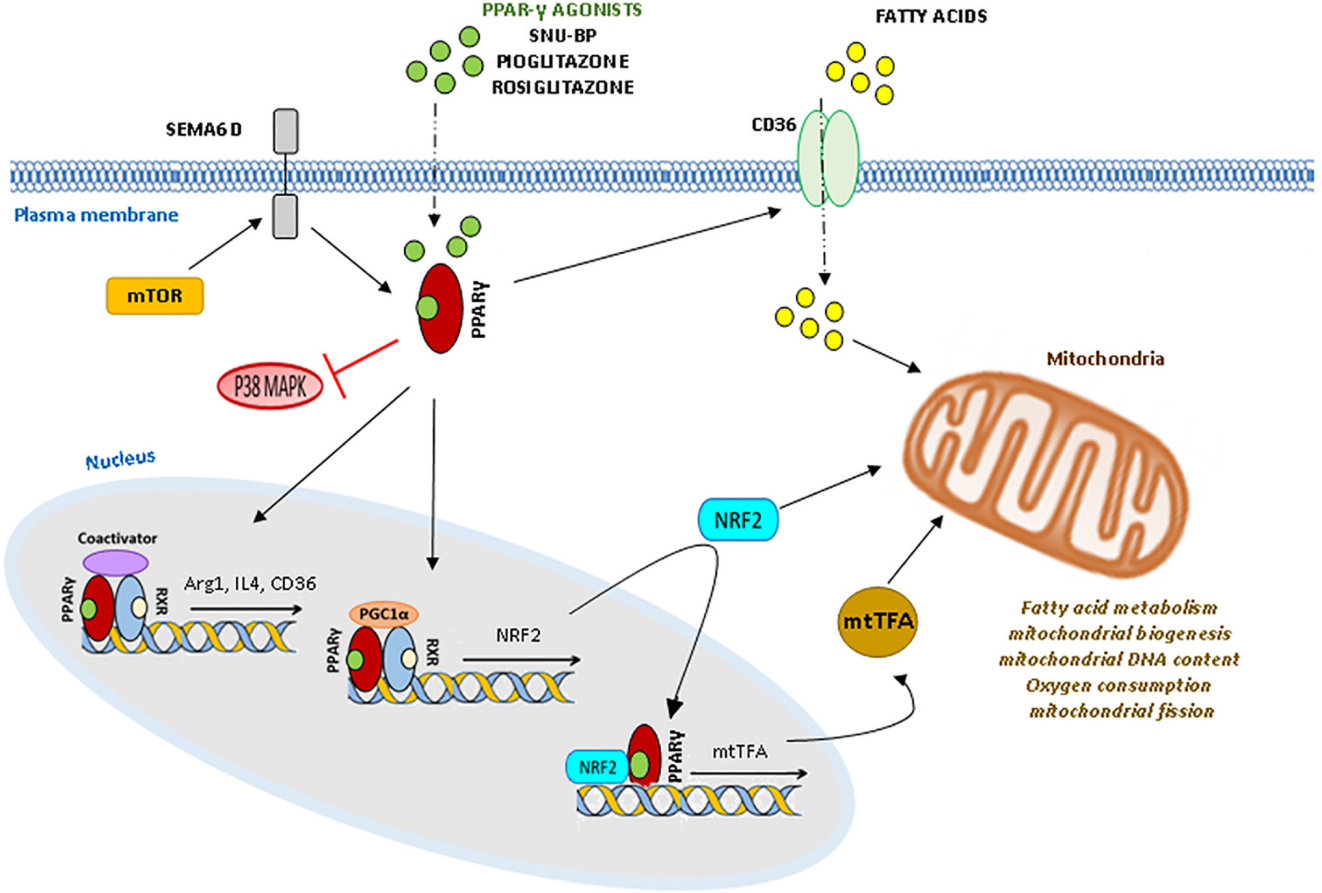
Intracellular receptors involved in beneficial microglia polarization. PPAR‐γ stimulation, by both natural and synthetic agonists (e.g., SNU‐BP, pioglitazone, rosiglitazone, malibatol A, galangin), inhibits expression of inflammatory mediators, while increase the expression of the anti‐inflammatory genes Arg‐1, IL‐4 and the fatty acid transporter CD36. Inhibition of p38‐MAPK, STAT‐1 and NF‐κB pathways are additional mechanisms underlying the anti‐inflammatory action of PPAR‐γ in microglia. As in other cell types PPARs‐γ may also interact with the PPAR‐γ coactivator 1‐alpha (PGC1α), the nuclear factor erythroid 2–related factor 1–2 (Nrf1–2), and mitochondrial transcription factors (mtTF)A, increasing mitochondrial functions. In addition in macrophages it mediates changes in lipid metabolism induced by mTOR kinase via Sema 6D

IL‐4 and IL‐13 are endogenous immune signals which polarize microglia toward neuron protective, pro‐regenerative phenotype. Despite microglia responsiveness to these cytokines have been studied mostly in culture, microglia have been shown to respond to IL‐4 administration with the expression of genes typical of alternative activation also in vivo (Pepe, Calderazzi, De Maglie, Villa, & Vegeto, 2014). The phenotypic features of IL‐4‐treated microglia are controlled by a network of molecular pathways that impact gene expression and cellular metabolism (Figure 1). Among them, the transcription factor STAT 6 accumulates in response to IL‐4 and, along with its downstream effector PPAR‐γ, has a central role in the regulation of transcription of anti‐inflammatory and pro‐resolving genes (Figure 1) (Lan, Han, Li, Yang, & Wang, 2017). The specific role of PPAR‐γ in driving positive metabolic and immune microglial functions will be discussed in a subsequent section. Additionally, IL‐4 induces expression of CD36, a receptor of lipoproteins, whose uptake and metabolism may support fatty‐acid oxidation, contributing to metabolic microglia reprogramming (Figure 2); (S. C. Huang et al., 2014).

IL‐10 drives microglia toward an alternative activated phenotype, which mainly participates in phagocytosis and removal of tissue debris (Chhor et al., 2013). IL‐10 suppresses pro‐inflammatory cytokine production from microglia via induction of suppressor of cytokine signaling (SOCS)1 and (SOCS)3 proteins. SOCS1,3 act in a negative feedback loop, inhibiting the cytokine‐activated Janus kinase (JAK)/STAT‐1 signaling pathway that are activated upon binding of pro‐inflammatory cytokines to their specific receptors (Figure 1).

Both IL‐4/IL‐13 and IL‐10 represent the challenging opportunities to convert microglia toward protective functions.

### 2.4 Triggering receptor expressed on myeloid cells‐2

Initial studies showed that protective microglial phenotype is induced by signals derived from apoptotic cells that activate the triggering receptor expressed on myeloid cells‐2 (TREM‐2). However, subsequent evidence (Krasemann et al., 2017) revealed that TREM‐2 activation by apolipoprotein E (APOE), the major risk factor for AD, identified as a TREM2 ligand (Atagi et al., 2015; Bailey, DeVaux, & Farzan, 2015), drives a neurodegenerative phenotype in microglia, characterized by suppression of major transcription factors of homeostatic microglia (PU.1, MEF2a, SMAD3, and TGF‐β signaling) and overlapping to some extent the classical pro‐inflammatory microglia phenotype, featured by high expression of the pro‐inflammatory miRNA miR‐155 (Butovsky et al., 2012). Thus, targeting of the TREM2‐APOE pathway might serve as a way to restore homeostatic microglia.

On the contrary, other studies revealed that TREM‐2 deficiency, by limiting microglia activation and phagocytosis, facilitates Aβ plaque buildup, and injury of adjacent neurons in mouse model of AD (Jay, von Saucken, & Landreth, 2017; Ulrich et al., 2014; Y. Wang et al., 2015; Y. Wang et al., 2016; Yuan et al., 2016) and results in impaired elimination of supernumerary synapses during brain development (Filipello et al., 2018). Interestingly the results coming from studies on TREM2‐deficient microglia showed reduced mitochondrial mass and increased phosphorylation of adenosine monophosphate‐activated protein kinase (AMPK) (Ulland et al., 2017), a key regulator of energy metabolism that is activated in response of low glucose and inhibits a shift in the cellular metabolism from oxidative phosphorylation to glycolysis (Hardie, 2011a, 2011b).

Importantly, these metabolic changes were reverted by supporting ATP synthesis, suggesting that microglia dysfunctions caused by TREM‐2 deficiency depend on energetic compromise (Ghosh, Castillo, Frias, & Swanson, 2017).

### 2.5 Potassium channels

The expression of Ca2^+^‐dependent K^+^ channel (KCa3.1) and K^+^ channels K_v_ 1.3 varies during microglia activation and can be a determinant for microglia polarization (Nguyen et al., 2017). KCa3.1 channels are voltage independent and only require a small increase in intracellular calcium to open. By maintaining a negative membrane potential through K^+^ efflux, the channels provide the driving force for prolong calcium influx which typically occurs during microglia respiratory burst (oxygen consumption by mitochondria) (Khanna, Roy, Zhu, & Schlichter, 2001), migration (Schilling, Stock, Schwab, & Eder, 2004), proliferation (Maezawa & Jin, 2010), and NO production (Maezawa & Jin, 2010), features of classical pro‐inflammatory microglia. Also the voltage‐gated potassium channel K_v_1.3 through K^+^ efflux helps to maintain a negative membrane potential (Maezawa et al., 2018), thus contributing to a pro‐inflammatory microglia phenotype. Both KCa3.1 and K_v_1.3 channels attracted interest as pharmacological targets for inhibiting detrimental microglia action in different pathological conditions, including spinal cord injury (Bouhy et al., 2011), ischemia (Y. J. Chen, Raman, Bodendiek, O'Donnell, & Wulff, 2011; Y. J. Chen et al., 2015), AD as well as cerebrovascular and traumatic brain injuries (Dale, Staal, Eder, & Moller, 2016; Maezawa et al., 2018; Maezawa, Jenkins, Jin, & Wulff, 2012; Nguyen et al., 2017). For example, KCa3.1 blockade with the small molecule TRAM‐34 has been shown to have beneficial effects in a rat MCAO ischemia model and to protect neurons from neurotoxicity caused by microglia activated with β‐amyloid (Aβ) oligomers (Figure 1) (Maezawa, Zimin, Wulff, & Jin, 2011). Similarly, the K_v_1.3 specific blocker PAP‐1 was recently shown to reduce neuroinflammation, Aβ plaque load and to improve behavioural deficits in a transgenic‐mouse model of AD, a disorder characterized by K_v_1.3 upregulation in microglia (Figure 1) (Maezawa et al., 2011; Rangaraju, Gearing, Jin, & Levey, 2015). Inhibition of p38‐mitogen‐activated protein kinase (MAPK), iNOS and cyclooxygenase 2 (COX‐2) expression likely accounts for beneficial effects of the both KCa3.1 and K_v_1.3 blockers (Figure 1) (Kaushal, Koeberle, Wang, & Schlichter, 2007; Nguyen et al., 2017).

### 2.6 Nuclear PPARs‐γ

As mentioned above, PPARs‐γ are nuclear receptors highly expressed in microglia, that play important roles in both the immune response and cell metabolism (Agarwal, Yadav, & Chaturvedi, 2017; Drew, Xu, Storer, Chavis, & Racke, 2006). Consolidated evidence indicates that PPAR‐γ activation by both natural and synthetic agonists, including the small molecule SNU‐BP, inhibits expression of surface antigen and synthesis of inflammatory mediators (Bernardo & Minghetti, 2006; Saijo, Crotti, & Glass, 2013; Q. Han et al., 2017; Y. Luo, He, Kuang, Jiang, & Yang, 2014), while increases the expression of the anti‐inflammatory genes Arg‐1 and IL‐4 (G. J. Song et al., 2016) and promotes microglial phagocytic ability (Figure 2).

PPARs‐γ have been implicated in the phenotypic switch induced in microglia by some neuroprotective natural compounds, such as malibatol A, a resveratrol agonist (Pan et al., 2015) and galangin, a molecule abundant in honey and medicinal herbs (Choi et al., 2017).

PPAR‐γ‐dependent microglia reprogramming toward beneficial function accounts for better outcome in several preclinical models of brain pathologies. The treatment of AD mice with the PPAR‐γ agonist pioglitazone, a drug used to treat type 2 diabetes, results in enhanced capability of microglia to phagocyte Aβ (Yamanaka et al., 2012) and cognitive improvement. Moreover, pioglitazone ameliorates the disease course in both the experimental autoimmune encephalomyelitis (EAE) model of MS (Feinstein et al., 2002; Storer, Xu, Chavis, & Drew, 2005) and in mice subjected to chronic mild stress (Q. Zhao et al., 2016).

PPARs‐γ regulate inflammatory pathway in microglia by several mechanisms. They block p‐38MAPK inflammatory pathways, resulting in decreased microglia reaction (Figure 2) (Ji et al., 2010) and reduce the activation of the classical pro‐inflammatory transcription factors STAT‐1 and NF‐κB, which are known to mediate both LPS and IFNγ inflammatory signaling in microglia (Bernardo & Minghetti, 2006). However, the pivotal role of PPARs‐γ in regulating the activation state of microglia may be due to metabolic reprogramming, as recently highlighted in macrophages, where PPAR‐γ has been implicated in mTOR‐dependent fatty acid uptake and lipid metabolic reprogramming, downstream activation of semaphorin 6D (Sema6D) (Kang et al., 2018), a key regulator of alternative macrophage polarization (Figure 2). Indeed, through interaction with downstream transcription factors and coactivators, PPARs‐γ regulate the expression of genes involved in glucose metabolism in mitochondria and fatty acid oxidation (Monsalve, Pyarasani, Delgado‐Lopez, & Moore‐Carrasco, 2013; Willson, Lambert, & Kliewer, 2001). Specifically, when activated by the full agonist pioglitazone, PPARs‐γ increase mitochondrial biogenesis, mitochondrial DNA content and oxygen consumption through interaction with the PPAR‐γ coactivator 1‐alpha (PGC1α), the nuclear factor erythroid 2–related factor 1–2 (Nrf1–2), and mitochondrial transcription factors (mtTF)A (Gleyzer, Vercauteren, & Scarpulla, 2005), regulating antioxidant gene expression (Liddell, 2017) (Figure 2). In addition, PPAR‐γ activation increases mithocondrial fission, which mediates removal of damaged mitochondria and plays an important role in the assembly of mitochondrial electron transport chain (Corona & Duchen, 2016).

Upstream PPAR‐γ expression, it has been identified msh‐like homeobox‐3 (MSX3), a homeobox gene particularly upregulated in pro‐regenerative microglia, that acts as pivotal inducer for pro‐regenerative microglial state (Z. Yu et al., 2015).

## 3 MOLECULAR MECHANISMS LINKING RECEPTOR/CHANNEL ACTIVATION TO THE BIOENERGETIC STATE OF MICROGLIA

**Figure 3:**
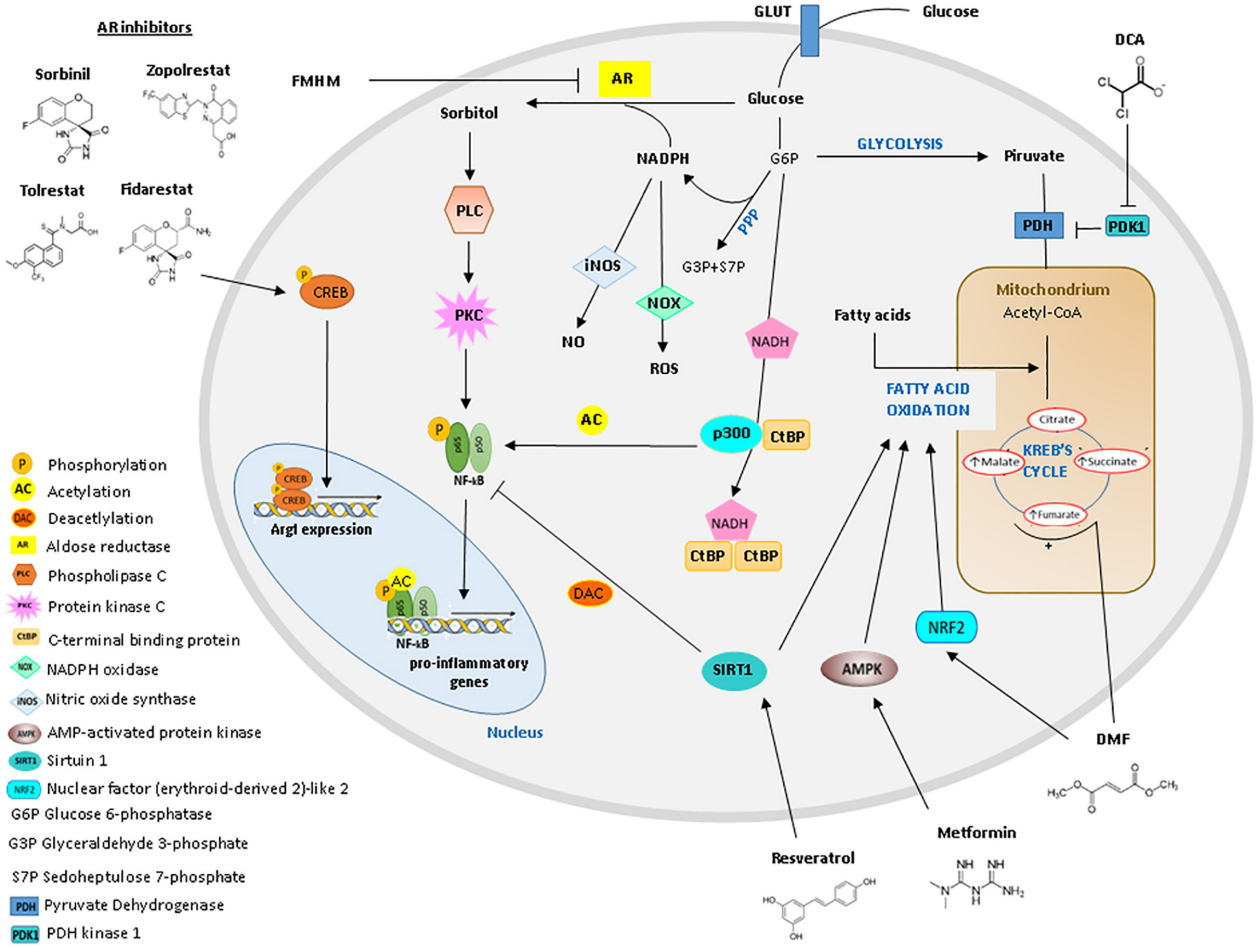
Metabolic drugs and molecular pathways exploitable to drive microglia into a pro‐regenerative phenotype. Dichloroacetate (DCA) promotes glucose metabolism in mitochondria by increasing the flux of pyruvate. Dimethylfumarate (DMF) promotes an antioxidant response and mitochondrial biogenesis through activation of the Nrf2 pathway. It may also enhance TCA cycle by increasing the TCA cycle intermediate fumarate. Metformin promotes oxidative phosphorylation through AMPK activation. Aldose reductase (AR) converts glucose into sorbitol using NADPH as a cofactor. Sorbitol, in turn, activates the phospholipase C/protein kinase C signaling pathway, resulting in downstream activation of pro‐inflammatory NF‐κB. By inhibiting AR, the small‐molecule FMHM, Sorbinil, Zopolrestat, and Fidarestat, limit inflammatory microglia response. Furthermore, Fidarestat induces CREB phosphorylation to increase Arg1. SIRT1 activators, such as Resveratrol, inhibit the pro‐inflammatory NF‐kB pathway and activate fatty acid metabolism. The scheme also depicts how NF‐kB, iNOS, and NOX activities are influenced by the bioenergetics state of microglia. NADPH generated via glucose metabolism through PPP is a cofactor for the production of NO, by iNOS, and of superoxide, by NOX. NADH generated through glycolysis promotes dimerization of C‐terminal binding protein (CtBP), preventing the corepressor activity of the monomeric form on the p300 acetylase, a NK‐kB activator, with consequent activation of NF‐kB transcriptional activity

Pharmacological analysis of microglia receptor/channel signaling cascades points to p‐NF‐κB, p38 ‐MAPK, iNOS, COX‐2, and NOX2 as key inflammatory intracellular pathways downregulated by agents which dampen microglia activation under pathological stimuli. Intriguingly, emerging evidence indicates that at least p‐NF‐κB, iNOS and NOX2 activity are influenced by the bioenergetics state of microglia, as clearly described in a recent review (Ghosh et al., 2017) (Figure 3). In fact, NADPH generated via glucose metabolism through the penthose phosphate pathway (PPP) is requisite cofactor for the production of NO, by iNOS, and of superoxide, by NOX. Moreover, glucose flux through glycolysis increases iNOS and pro‐inflammatory gene expression via NADH production (Shen et al., 2017). Specifically, NADH promotes dimerization of C‐terminal binding protein (CtBP), a transcriptional corepressor which normally binds and inhibits p300 acetylase with resultant release of p300 from CtBP. Unbound p300 acetylates the p65 subunit of NF‐κB and promotes its inflammatory transcriptional activity (Figure 3). By these molecular mechanisms glucose metabolism via glycolysis and PPP can be a determinant of the pro‐inflammatory microglia state. In support to this hypothesis, inhibition of glycolytic metabolism has been recently shown to abolish the inflammatory state of microglia lacking Clk1 (R. Gu et al., 2017), as already mentioned in the introduction paragraph.

The link between glucose metabolism in the cytoplasm outside mitochondria and inflammation implicates that agents reprogramming microglia toward protective functions would decrease glucose flux through both glycolysis and the PPP in order to downregulate iNOS and NOX2. This may cause a compensatory increase in glucose‐based energy production in mitochondria and fatty acid‐based energy production. Consistent with this hypothesis, PPAR‐γ signaling pathway, that is induced by agents which redirect microglia toward protective functions, promotes biogenesis of mitochondria and mitochondrial glucose metabolism, as mentioned above, and promote fatty acids beta oxidation, linking pro‐regenerative microglia phenotype to fatty acid‐ and glucose‐based energy production in mitochondria (Figure 2).

## 4 ALIMENTARY COMPONENTS DRIVING PRO‐REGENERATIVE MICROGLIA FUNCTION THROUGH METABOLIC REMODELING

Bioactive fatty acids derived from nutrition such as saturated (SFA) or polyunsaturated fatty acids (PUFA) can cross the blood–brain barrier, and directly influence microglia, as recently reviewed by (Nadjar, Leyrolle, Joffre, & Laye, 2017).

While SFA mainly act as pro‐inflammatory stimuli (Z. Wang et al., 2012; Gao et al., 2014), N‐3 PUFAs and their metabolites have emerged as anti‐inflammatory modulators of microglial functions (Laye, Nadjar, Joffre, & Bazinet, 2018). Independent of the inflammatory challenge applied (e.g., hypoxia, interferon‐y, amyloid‐β), cultured microglia consistently show decreased production of pro‐inflammatory factors (De Smedt‐Peyrusse et al., 2008), decreased COX2 and iNOS activity (Pettit, Varsanyi, Tadros, & Vassiliou, 2013; Zendedel et al., 2015) while exibit typical features of anti‐inflammatory microglia, like CD206 surface expression and autophagy when treated with n‐3 PUFAs (Chhor et al., 2013; Inoue et al., 2017). Many in vivo studies confirm that N‐3 PUFA supplementation reduces detrimental microglia function (summarized in (Laye et al., 2018). Although microglia express a wide range of lipid‐sensitive receptors and lipid metabolism‐related genes (Mauerer, Walczak, & Langmann, 2009; Mecha et al., 2015), the molecular link between bioactive fatty acids and their effects in microglia has not been completely clarified yet. Emerging evidence suggests that PUFAs can drive a protective phenotype through the activation of CB2, which is among microglial receptors reprogramming microglia toward beneficial function (Guida et al., 2017; Mecha et al., 2015; Nunez et al., 2004) but other receptors may be involved including ALX/FPR2 receptors (Bisicchia et al., 2018) (Figure 1).

Molecular pathways for the anti‐inflammatory effects of N‐3 PUFAs include activation and overexpression of the protein deacetylase sirtuin1 (SIRT1), with subsequent suppression of NF‐κB via subunit p65 deacetylation (Figure 3); (Inoue et al., 2017). Inhibition of p38MAPK inflammatory pathway and PPAR‐γ activation are also in part responsible of protective effects of PUFAs and their products (Antonietta Ajmone‐Cat et al., 2012; De Smedt‐Peyrusse et al., 2008). Given the connection of NF‐κB and PPAR‐γ to the bioenergetics state of microglia (Ghosh et al., 2017), also dietary lipids may likely shape microglia phenotype acting on cell metabolism. In support to this hypothesis, fasting and ketogenic diet, that lead to a sustained reduction in blood glucose levels and to an increase in circulating ketones, have been reported to have anti‐inflammatory actions and suppress activation of microglia by regulating their metabolic features (Longo & Mattson, 2014). These effects have been shown to rely on the activation of the metabolite receptor GPR109A by B‐hydroxybutyrate, that attenuates NF‐κB signaling and pro‐inflammatory cytokine production (Fu et al., 2015) (Figure 1).

Other groups of bioactive compounds, normally present in foods, especially in the Mediterranean diet, such as phenolic compounds, phytosterols and carotenoids (e.g., lycopene, fucoxanthin, and lutein) exhibit anti‐inflammatory properties on microglia (Pena‐Altamira et al., 2017; Jeong et al., 2010; Sachdeva & Chopra, 2015; D. Zhao, Kwon, Chun, Gu, & Yang, 2017), but the mechanisms underlined their effects still remain to be defined.

## 5 MicroRNAs PROMOTING PRO‐REGENERATIVE MICROGLIA PHENOTYPE AND THEIR POTENTIAL LINK WITH ENERGY METABOLISM

MicroRNAs (miRNAs) are endogenous, short, noncoding RNAs that act as important posttranscriptional regulators of gene expression, playing crucial roles in both the brain (Cao, Li, & Chan, 2016; Schratt, 2009) and the immune system (Xiao & Rajewsky, 2009). Due to their ability to simultaneously modulate the fate of different genes, these molecules are particularly well suited to act as key regulators during neural and immune cell differentiation and activation.

The impact of miRNAs on microglial phenotype has been recently revealed by generating mice lacking microglial Dicer, an enzyme necessary for miRNA maturation. Dicer ablation resulted in hyper‐responsiveness of adult microglia to stimuli (peripheral endotoxin), indicating that miRNAs overall limit microglia responses to challenge (Varol et al., 2017). A number of studies dissected the role of specific miRNAs in microglia (Guedes, Cardoso, & Pedroso de Lima, 2013; Karthikeyan, Patnala, Jadhav, Eng‐Ang, & Dheen, 2016; Ponomarev, Veremeyko, & Weiner, 2013). Given the central role of cellular metabolism in the effector function of microglia, we focus below on anti‐inflammatory miRNAs induced in response to either pro‐ or anti‐inflammatory stimuli, which switch microglia toward protective functions and that may act by modulating bioenergetic metabolism (listed in Table 1).

**Table 1:** MicroRNAs with proregenerative functions in microglia and their metabolic targets

miRNA	Microglia pro‐regenerative function	Reference	Metabolictarget gene	Metabolic pathway	Model	Reference
miR‐124	Downregulates IL‐6, TNF‐α, iNOS	Sun et al., 2013; Ponomarev et al., 2011; Huang et al., 2018	RPIA	Pentose phosphate shunt	Human colorectal cancer cells	Qiu Z. Et al., 2015
	Increases TGF‐β, arginase‐1, and FIZZ1	Ponomarev et al., 2011	PRPS1, PDK1	Lactate production	Human colorectal cancer cells	Qiu Z. Et al., 2015
	Reduces motility and phagocytosis capacity	Svahn et al., 2015; Pinto et al., 2017				
	Targets C/EBPa	Zhang P et al., 2004; Yu A 2017				
	Supresses p38‐MAPK	Lawson SK et al., 2013				
miR‐200b	Reduces iNOS expression and NO production	Jadhav SP et al., 2014	LDHA	Aerobic glycolysis	Glioma cells	Hu S et al., 2016
	Suppresses c‐Jun and JNK activity					
	Reduces the migratory ability					
miR‐146a	Targets the key inflammatory regulators IRAK1/2 and TRAF6	Jayadev et al., 2013; Sharma N et al., 2015	Undefined	Glycolysis	Synovial fibroblasts	Saferding V 2017
	Negatively regulates IL‐6 and TNF‐α	Zhao H et al., 2017	FAS	Fatty acid synthesis	Mesenchymal stem cells; germinal center B cells	Suzuki Y et al.,2010; Guo et al., 2013
					
miR‐223	Increases Arg1 and IL‐10 expression	Wei Ying et al., 2015	Undefined	Glycolysis	Macrophages	Zhuang G et al., 2012
	Suppresses PKNOX1, NFAT and RASA1					
	It is required for PPARγ function	Zhuang G, 2012				
miR‐181a	Down‐regulates IL‐1α	Xie et al., 2013	IDH1	NADPH production	Mouse embryonic fibroblasts	Chu B et al., 2015
	Inhibits levels of IL‐1b, IL‐6, and TNFa			Oxidative metabolism	Colon cancer cells; hepatocytes	Wei Z et al., 2014; Du X et al., 2017
	Targets C/EBPα and KLF6	Jia Bi et al., 2016				
	Increases PPARy levels					
miR‐let‐7	Inhibits INOS and IL‐6 expression	Cho KJ et al., 2015	PDK1, insulin‐PI3K‐mTOR pathway, EZH2, IRS2	Glucose uptake and lactate production	Human hepatocellular carcinoma, C2C12 myoblasts, human embryonic stem cell‐derived cardiomyocytes	Ma X et al., 2014; Zhu H et al., 2011; Kuppusamy KT et al., 2015
	Reduces ROS, and enhances IL‐10, IL‐4		Ppargc1b	Gluconeogenesis, beta‐oxidation of fatty acids and ketogenesis during fasting	Human adipose‐derived mesenchymal stem cells	Wei J et al. 2014
	Targets PAK and C/EBPα	Banerjee et al., 2013; Zhang W et al., 2015				
	Suppresses the release of inflammatory mediators	Lv J et al., 2018				
miR‐21	Suppresses IRAK and MyD88 and PDCD4	Chen et al., 2013; Sheddy et al., 2010	PEPCK; G6Pase, FOXO1	Glycolysis	Hepatocytes	Luo et al., 2017
	Negatively regulates TLR‐4 signaling	Fafian‐Labora J et al., 2017				
	Suppresses FasL expression	Zhang L, et al. 2012				
miR‐29b	Suppresses immune responses to intracellular pathogens by targeting IFN‐k	Ma et al., 2011	PPARδ, SPARC	Glycose uptake	Skeletal muscle cells, adipocytes	Zhou Y et al., 2016; song H 2018
			IRS1, PI3K, and AKT2	Glycolysis	Human and mouse skeletal muscle cells	Massart J et al., 2017
			PPARD	Fatty acid oxidation	Human and mouse skeletal muscle cells	
miR‐125a	Not defined		ENO‐1, HK2, PFK1	Glycolysis	Hepatocellular carcinoma cells	Jin et al., 2017

MiR‐124 was one of the first miRNA to be linked to pro‐regenerative microglial phenotype (Y. Sun, Luo, Guo, Su, & Liu, 2015). It is absent in perinatal microglia but present at high levels in adult microglia where it is strongly downregulated under inflammatory conditions (~70% decrease in EAE mice) (Ponomarev, Veremeyko, Barteneva, Krichevsky, & Weiner, 2011). Its forced expression downregulates pro‐inflammatory markers, while increases the expression of protective markers, as is the case of TGF‐β, Arg1, and FIZZ1 (Y. Sun et al., 2013; Ponomarev et al., 2011; S. Huang et al., 2018; Periyasamy et al., 2018) and reduces microglial phagocytic ability (Pinto, Cunha, Barbosa, Vaz, & Brites, 2017; Svahn, Giacomotto, Graeber, Rinkwitz, & Becker, 2016). The contribution of miR‐124 to homeostatic/anti‐inflammatory microglia functions relies on silencing of CCAAT/enhancer‐binding protein (C/EBP)‐α (P. Zhang et al., 2004), one of the major transcription factor that drives pro‐inflammatory microglia polarization (A. Yu et al., 2017). Moreover, miR‐124 suppresses p38‐MAPK, that also has central role in coordinating inflammatory microglia responses (Lawson et al., 2013). Of relevance for metabolic microglia reprogramming, miR‐124 (Qiu et al., 2015) directly controls the bioenergetic state of the cells. Specifically, it reduces lactate production and the PPP, that supports NO and superoxide production, by targeting mRNAs encoding phosphoribosyl pyrophosphate synthetase 1 (PRPS1) and ribose‐5‐phosphate isomerase‐A (RPIA) (Qiu et al., 2015); (Table 1).

MiR200b reduces iNOS expression and NO production and limits the migratory potential of reactive microglia. Suppression of the glycolytic enzyme lactate dehydrogenase A (LDHA) and aerobic glycolysis likely underlies the miRNA action (S. Hu et al., 2016); (Table 1).

MiR‐146a is a microglia‐enriched miRNA (Jovicic et al., 2013) induced in inflammatory microglia, which dampens inflammation by suppressing the key inflammatory proteins interleukin‐1 receptor associated kinase 1 (IRAK1) and TNF receptor associated factor‐6 (TRAF‐6) (Jayadev et al., 2013; Sharma, Verma, Kumawat, Basu, & Singh, 2015). In addition, miR‐146a may promote microglia transition toward pro‐regenerative traits by enhancing mitochondrial energy metabolism and fatty acid oxidation. This intriguing hypothesis is supported by recent evidence showing (a) miR‐146a‐dependent PPAR‐γ upregulation at both protein and mRNA levels (C. Huang et al., 2016); (b) positive correlation between circulating levels of miR‐146a and metabolic TCA cycle intermediates, which are indicative of intense glucose metabolism in mitochondria (Wu et al., 2015); and (c) the ability of the miR‐146a to inhibit glycolytic activity (Saferding et al., 2017) and to reduce fatty acid synthesis, via FASN silencing (Guo et al., 2013; Suzuki, Kim, Ashraf, & Haider, 2010) (Table 1).

MiR‐223 is another microglia‐enriched miRNA (Jovicic et al., 2013; Prada et al., 2018) that promotes anti‐inflammatory gene expression (Table 1). Its deficiency leads to compromised pro‐regenerative differentiation in response to IL‐4 (Zhuang et al., 2012). Three important inflammatory genes are validated miR‐223 targets: the pro‐inflammatory regulator PBX/knotted 1 homeobox 1 (Pknox1) (Zhuang et al., 2012), the transcription factor NFAT5, that is involved in NF‐κB signaling (Lopez‐Rodriguez et al., 2001) and RAS p21 protein activator 1 (RASA1), a crucial component of pro‐inflammatory pathways in macrophage (Ying et al., 2015). Importantly miR‐223 has been shown to be required for PPAR‐γ function (Zhuang et al., 2012), linking the acquisition of pro‐regenerative traits to mitochondrial glucose metabolism and fatty acid beta oxidation.

miR‐181a targets the inflammatory genes IL‐1α (Xie et al., 2013), TNF (He et al., 2013) and the transcriptional factor C/EBPα but also suppresses Kruppel‐like factor 6 (KLF6) (Bi et al., 2016), a PPAR‐γ inhibitor (Date et al., 2014), thus likely favoring PPAR‐γ‐dependent energy metabolism in mitochondria and fatty acid peroxidation in microglia. This has been proven in lymphocytes where miR‐181a enhances the expression of genes involved in beta oxidation while suppresses isocitrate dehydrogenase 1 (IDH1), a cytoplasmic enzyme involved in production of NADPH, the cofactor for NO and superoxide generation (Lian et al., 2016; Chu, Wu, Miao, Mei, & Wu, 2015; Table 1).

let 7b belongs to the let 7 family of miRNAs, that dampens production and release of inflammatory molecules from microglia (Table 1). p21‐activated kinase 1 (PAK) and C/EBP have been disclosed as downstream inflammatory targets of let 7 miRNAs (Banerjee, Xie et al., 2013). However, let 7 miRNAs also serve as important mediators in energetic metabolism (Chu et al., 2015). They reduce glucose uptake and lactate production and prompt the use of fatty acid as energy source. However, further work is required to define how let 7b impacts microglia metabolism.

MiR‐21 is one of the most common anti‐inflammatory miRNAs (Sheedy, 2015). It is a negative regulator of TLR‐4 signaling (Fafian‐Labora et al., 2017), able to downregulate the expression of two essential mediators for NF‐κB activation, the TLR‐4 adaptor protein MyD88 and its downstream target IL1 receptor‐associated kinase (IRAK) (Y. Chen et al., 2013). In addition, miR‐21 targets programmed cell death protein 4 (PDCD4) (Sheedy et al., 2010) and the apoptotic protein FasL (L. Zhang, Dong, Li, Hong, & Wei, 2012) protecting against microglia‐mediated neuron cell death in hypoxic/ischemic conditions (L. Zhang et al., 2012). Despite the action on miR‐21 on microglia bioenergetic state is still undefined, miR‐21 inhibits glucose production by targeting phosphoenolpyruvate carboxykinase (PEPCK), glucose‐6‐phosphatase (G6Pase), and forkhead box protein O1 (FOXO1) an important transcription factor of insulin signaling (A. Luo et al., 2017); (Table 1).

miR‐29b is upregulated in cultured microglia during neuroviral infection (Thounaojam, Kaushik, Kundu, & Basu, 2014) and suppresses immune responses to pathogens by targeting interferon‐k (IFN‐k) (Ma et al., 2011). As other member of the miR‐29 family, miR‐29b reduces glucose uptake (Zhou et al., 2016; H. Song, Ding, Zhang, & Wang, 2018) and glycolysis (Massart et al., 2017). However, whether miR‐29 impacts glucose metabolism in a similar manner in microglia remains still unknown.

Finally, miR‐125‐a, known to be involved in macrophage differential activation through downregulation of TNF‐α‐induced protein 3 (TNFAIP3) (Graff, Dickson, Clay, McCaffrey, & Wilson, 2012) and of the inflammatory transcription factor Kruppel‐like factor 13 (KLF13) (Banerjee, Cui, et al., 2013), has been recently found to be upregulated in reactive microglia and in extracellular vesicles (EVs) thereof (Prada et al., 2018). Few glycolytic enzymes, including enolase‐1 (ENO‐1), HK2 (F. Jin et al., 2017; C. M. Sun, Wu, Zhang, Shi, & Chen, 2017) and phosphofructokinase (PFK1) are miR‐125‐a validated targets (Table 1). Consistently, miR‐125‐a decreases the uptake of glucose and the production of lactate, and the levels of ATP and ROS in other cell types (F. Jin et al., 2017). Whether the miRNA suppresses glycolysis in microglia remains to be proven.

## 6 AN ATTRACTIVE CELL THERAPY APPROACH TO REDIRECT MICROGLIA TOWARD BENEFICIAL FUNCTIONS: INVOLVEMENT OF ENERGY METABOLISM

Mesenchymal stem cells (MSCs) are widely used in cell therapy of brain injury and inflammation thanks to their immunomodulatory properties, their self‐renewal, their multipotency and their minimally invasive accessibility that make them an attractive tool for regenerative medicine. A large part of MSC positive effects across models of brain injury is due to their secretion products which create a favorable regenerative microenvironment. MSC secretome includes soluble factors, released in a naked form, and molecules (proteins, lipids and genetic material) released in association with EVs. EVs, including exosomes and microvesicles, are fundamental mediators of intercellular communication. They shuttle bioactive molecules from one cell to another, causing the exchange of information and reprogramming of the recipient cells (Turola, Furlan, Bianco, Matteoli, & Verderio, 2012).

Previous findings from our group and others have shown that through their secretome MSCs re‐direct microglia from detrimental toward pro‐regenerative functions (Giunti et al., 2012; Zanier et al., 2014). By investigating post‐traumatic changes of microglia in mice that had received MSCs through intracerebroventricular administration, we observed an increased number of Ym1(+) protective microglia, associated with early and persistent recovery of neurological functions. MSCs, indirectly co‐cultured with microglia in vitro*,* directly counteract the pro‐inflammatory response of cells activated with inflammatory cytokines and induce persistent pro‐regenerative traits (Giunti et al., 2012; Zanier et al., 2014). More recent studies demonstrated that MSCs‐derived EVs may be sufficient to significantly dampen microglia response and improve functional recovery upon intravenous administration in traumatic brain or spinal cord injuries (L. X. Zhang, Yin, Zhang, & Deng, 2015 *;* J. W. Kim, Ha, Molon, & Kim, 2013; Ruppert et al., 2018) as well as in a mouse model of AD. Despite these findings offer a hopeful opportunity to advance novel cell‐free therapeutic strategies that might prevail over the risks associated with the use of cells, a still unresolved issue is the molecular mechanism whereby the infused EVs reduce inflammation and rescue cognitive impairments in rodent models of neuroinflammatory diseases. Indeed, EVs contain many active components and it is difficult to identify those with significant effects on microglia and other brain cells (D. K. Kim et al., 2016).

An alternative source of stem cells able to redirect brain inflammatory myeloid cells (microglia and monocyte‐derived cells) are neural precursor cells (NPCs) (De Feo et al., 2017). Martino and coworkers recently showed that intrathecal transplantation of NPCs in mice with EAE restrains microglia activation and impairs the accumulation of inflammatory monocyte‐derived cells, favoring their switch toward an anti‐inflammatory phenotype. Transcriptome analysis, combined to the use of TGF‐β2 knockout neural progenitors, identified a secretory protein, TGF‐β2, as the primary factor responsible for NPC immunomodulation and disease amelioration. TGF‐β2 is a well‐known inducer of Arg1*,* the key metabolic enzyme which distinguishes alternatively activated myeloid cells (Gordon, 2003). Consistent with the presence of TGF‐β responsive elements in the flanking region of the metabolic gene (Peranzoni et al., 2007), Martino and colleagues found by transcriptome analysis an early upregulation of Arg1 in myeloid cells exposed in vitro to NPCs, followed by subsequent induction of anti‐inflammatory molecules (e.g., Hspa1a, IL‐1 decoy receptor that blocks IL‐1 [Il1r2] and the NF‐κB inhibitor Ascc1). Moreover, a profound rearrangement of metabolic and synthetic processes was observed in vivo in myeloid cells FACS‐sorted from EAE mice intrathecally injected with NPCs. In particular, oxidative phosphorylation was the most prominent pathway altered under NPC treatment.

Very recently a significant progress has been made toward understanding the molecular mechanism that underpins the capacity of NPCs to drive pro‐inflammatory microglia and infiltrating macrophages toward beneficial functions in EAE (Peruzzotti‐Jametti et al., 2018). Pluchino and colleagues clearly showed that NPCs counteract the metabolic changes of pro‐inflammatory cells and reprogram them toward an oxidative phosphorylation anti‐inflammatory phenotype. Indeed NPCs restore basal oxygen consumption rate and extracellular acidification rate in pro‐inflammatory macrophages. Furthermore, by performing an untargeted mass spectrometry analysis of the extracellular and intracellular metabolite content of macrophages, they identified the metabolite succinate as the main target of the pro‐regenerative NPC action. Intracellular succinate is known to act as a key pro‐inflammatory signal in phagocytes, by enhancing IL‐1β generation (Littlewood‐Evans et al., 2016; Tannahill et al., 2013) and favouring mitochondrial production of ROS over ATP synthesis (E. L. Mills et al., 2016). Extracellular succinate also exhibits pro‐inflammatory activity though interaction with its specific receptor SUCNR1 and it is emerging as a biomarker of metabolic distress and inflammatory activity (Littlewood‐Evans et al., 2016; E. Mills & O'Neill, 2014). Specifically, the work by Pluchino and colleagues demonstrated that succinate is released by inflammatory phagocytes and activates SUCNR1 on NPCs, leading to the acquisition of an anti‐inflammatory NPC phenotype. In fact, in NPCs SUCNR1 activation elicits both secretion of the immune modulator PGE2, that in turn deactivates pro‐inflammatory phagocytes, and upregulation of two succinate co‐transporters (i.e., SLC13A3 and SLC13A5) which remove the extracellular metabolite and prevent its autocrine and paracrine action on inflammatory phagocytes. A significant decrease in succinate concentration was reported in the CSF of EAE mice transplanted with NPCs and the expression of SUCNR1 was shown to be necessary for the therapeutic effects of transplanted NPCs in vivo. Besides identifying scavenging of extracellular succinate as a novel anti‐inflammatory mechanism underlying the regenerative potential of NPCs, this work provides a direct link between NPC‐dependent phenotypic and metabolic switch in microglia/macrophages.

## 7 METABOLIC DRUGS AND MOLECULAR PATHWAYS WHICH MAY BE EXPLOITED TO DRIVE MICROGLIA INTO A NEURO‐SUPPORTIVE PHENOTYPE

Some metabolic pathways may be exploited to drive a pro‐regenerative microglia phenotype, and a few drugs may already be available for metabolic microglia reprogramming (Table 2).

**Table 2:** Metabolic drugs for pro‐regenerative microglia polarization

Targets	Drugs	References
PDK 1 (inhibitor)	DCA	Kato et al., 2007; Gerriets et al., 2015
Nrf2 (activator)	DMF	Liddell, 2017
AMPK (activator)	Metformin	Hardie, 2011a, 2011b
PPARγ (agonist)	Pioglitazone	Heneka et al., 2015
	Rosiglitazone	Heneka et al., 2015
	DSP‐8658	Yamanaka et al., 2012
	MDG548	Lecca et al., 2015
	SNU‐BP	Bernardo & Minghetti, 2006
Aldose reductase (inhibitor)	Small‐molecule FMHM	Zeng et al., 2013
	Sorbinil	X. M. Song et al., 2017
	Zopolrestat	X. M. Song et al., 2017
	Fidarestat	Q. Zhang et al., 2016
	Tolrestat	Rosa & Dias, 2014
Sirtuins (activator)	Resveratrol	Carafa et al., 2016

Dichloroacetate (DCA) is a small orally available drug with several therapeutic applications including MS, solid tumors and inherited mitochondrial disorders (James et al., 2017) based on its ability to inhibit the kinase PDK1 and to consequently increase pyruvate dehydrogenase (PDH) activity and the flux of pyruvate into the mitochondria (Gerriets et al., 2015; Kato, Li, Chuang, & Chuang, 2007); (Figure 3). DCA has been shown to shifts glucose metabolism toward the TCA cycle/oxidative phosphorylation in T cells in mice affected by EAE (Gerriets et al., 2015) and to promote a pro‐regenerative microglia/macrophages phenotype in vitro and in a peripheral inflammation model (Kato et al., 2007). However, whether DCA shifts microglial phenotype by promoting oxidative phosphorylation has not been proven yet.

Dimethylfumarate (DMF) is an approved drug for MS, known to switch pro‐inflammatory microglia to a neuroprotective state (Parodi et al., 2015) and to reduce disease progression via activation of (erythroid‐derived 2)‐related factor‐2 (Nrf2), a transcription factor with antioxidant properties (Liddell, 2017). DMF has been proposed to exert its therapeutic effects modulating oxidative phosphorylation, through increased levels of the TCA cycle intermediate fumarate in microglia (Tannahill, Iraci, Gaude, Frezza, & Pluchino, 2015); (Figure 3). However, this intriguing hypothesis still remains to be validated.

Metformin is a widely used drug to treat diabetes and a well‐known activator of AMPK, already mentioned to be activated in response of low glucose and to promote oxidative phosphorylation (Hardie, 2011a, 2011b); (Figure 3) Metformin has been recently shown to promote pro‐regenerative microglial phenotype and significantly ameliorate neurobehavioral function after stroke (Q. Jin et al., 2014) and traumatic brain injury (Tao et al., 2018), suggesting that it may be used to enhance pro‐regenerative microglial function by promoting oxidative metabolism.

PPAR‐γ activation is an alternative way to push oxidative metabolism in microglia, as already mentioned. PPARs‐γ can be activated with thiazolidinediones (e.g., pioglitazone or rosiglitazone) a class of antidiabetic drugs proven to be effective in reducing the extent of neuroinflammation in different models of brain diseases and to attenuate neurodegeneration in patients with mild‐to‐moderate dementia (Heneka, Fink, & Doblhammer, 2015).

Protective microglial function may be also promoted by pharmacological inhibitors of aldose reductase (AR), a metabolic enzyme implicated in inflammation‐related diseases, that converts glucose into sorbitol using NADPH as a cofactor (Petrash, 2004); (Figure 3). The small‐molecule FMHM, a natural derived AR inhibitor, was initially reported to suppress the expression of various inflammatory genes in microglia both in vitro and in vivo. Mechanistically FMHM suppresses the activity of AR‐dependent phospholipase C/protein kinase C signaling, resulting in downstream inactivation of NF‐κB inflammatory pathway (Zeng et al., 2013); (Figure 3). More recently, two other AR inhibitors have been identified, Sorbinil and Zopolrestat, which significantly inhibit inflammatory cytokine expression by blocking NF‐κB and MAPK signaling pathways in Aβ‐treated microglia (X. M. Song et al., 2017); (Figure 3). In support to these findings, Zhang and colleagues showed that AR deficiency drives microglia/macrophages toward an anti‐inflammatory after spinal cord injury in mice and treatment with the AR inhibitor Fidarestat induces c‐AMP response element binding protein (CREB) phosphorylation to increase Arg1 expression in cultured microglia (Figure 3); (Q. Zhang et al., 2016). Importantly some AR inhibitors have gone through Phase‐3 clinical studies (Pandey, Srivastava, & Ramana, 2012) and Tolrestat has been approved to prevent diabetic complications (Rosa & Dias, 2014). Finally, regulation of sirtuins (SIRTs) may represent another strategy to promote protective microglial phenotype through metabolic remodeling. SIRTs are a family of NAD‐dependent lysine deacetylases, that play a key role in regulation of cellular metabolism among other fundamental cell function. SIRT1 and SIRT6 were reported to coordinate a switch from glycolysis to fatty acid oxidation in macrophages (J. Yu & Auwerx, 2009) and SIRT1 and 2 were shown to prevent excessive microglia activation through NF‐κB deacetylation (Figure 3); (L. Li et al., 2015); (Inoue et al., 2017; Pais et al., 2013). Starting from Resveratrol, the first SIRT1activator described, other SIRT activators have been developed and some of them are currently in clinical trials for the treatment of age‐related neuroinflammation (Carafa et al., 2016).

## 8 CONCLUSIONS

A large variety of receptors are present in microglia, whose activation by immune signals, neurotransmitters, neuropeptides, hormones, lipid messengers, metabolites, and even alimentary components drives microglia toward beneficial functions. Their signaling pathways converge on common anti‐inflammatory and pro‐regenerative molecular programs. While the nuclear receptor PPAR‐γ, that promotes glucose and fatty acid metabolism in mitochondria, is consistently induced, the inflammatory pathways dependent on NF‐κB, p38‐MAPK, iNOS, COX‐2 and NOX2 are commonly inhibited. Importantly, iNOS, NOX2 and NF‐κB activities are strictly connected to glucose metabolism through glycolysis and the PPP, implicating that a change in microglia bioenergetics would shape the microglia phenotype. Specifically switching from aerobic glycolysis to oxidative phosphorylation would dampen microglia pro‐inflammatory activity.

A set of miRNAs with anti‐inflammatory action has been identified. Among their validated target genes, we here highlighted many metabolic enzymes involved in glucose uptake, glycolysis, lactate production, pentose phosphate shunt, suggesting a connection between miRNA‐induced transition toward protective phenotype and changes in energy metabolism.

Finally stem cells, (at least NPCs), which redirect microglia toward protective function, induce a profound rearrangement of metabolic and synthetic microglial processes, further supporting the key involvement of bioenergetics in microglia reprogramming toward an alternative activation state.

From this scenario, targeting cell metabolism emerges as a new potential therapeutic approach to redirect microglia from detrimental to pro‐regenerative phenotype. In this respect, two drugs shifting glucose metabolism toward the TCA cycle/oxidative phosphorylation (DCA and DMF) have already been approved for the treatment of MS, the prototypical neuroinflammatory disease characterized by microglia inflammation and drugs approved to treat metabolic disorders (metformin and thiazolidinediones) have been proven to dampen excessive microglial activation in brain diseases.

## CONFLICT OF INTEREST

The authors declare that they have no conflict of interest.

## AUTHOR CONTRIBUTIONS

All authors: conception of idea, review of the literature, manuscript writing and editing.
